# Association between Serum Testosterone and Aortic Valve Stenosis: A Prospective Cohort Study

**DOI:** 10.3390/jcdd10110454

**Published:** 2023-11-09

**Authors:** Jari A. Laukkanen, Carl J. Lavie, Setor K. Kunutsor

**Affiliations:** 1 Institute of Public Health and Clinical Nutrition, University of Eastern Finland, P.O. Box 1627 Kuopio, Finland; 2 Institute of Clinical Medicine, Department of Medicine, University of Eastern Finland, P.O. Box 100 Kuopio, Finland; 3 Wellbeing Services County of Central Finland, Department of Medicine, 40620 Jyväskylä, Finland; 4 Ochsner Heart and Vascular Institute, Ochsner Clinical School-The University of Queensland School of Medicine, New Orleans, LA 70121, USA; clavie@ochsner.org; 5 Diabetes Research Centre, Leicester General Hospital, University of Leicester, Leicester LE5 4PW, UK; skk31@cantab.net

**Keywords:** testosterone, aortic stenosis, cohort study

## Abstract

Serum testosterone is associated with atherosclerotic cardiovascular disease, which shares risk factors with aortic stenosis (AS). The association between serum testosterone and AS has not been previously investigated. We aimed to assess the prospective association between serum testosterone and risk of AS. Serum testosterone was determined at baseline using a radioimmunoassay kit in 2577 men aged 42–61 years recruited into the Kuopio Ischemic Heart Disease prospective cohort study. Hazard ratios (HRs) with 95% confidence intervals (Cis) were estimated for AS. After a median follow-up of 27.2 years, 119 cases of AS were recorded. The risk of AS increased continuously with increasing serum testosterone across the range 25–39 nmol/L (*p*-value for nonlinearity = 0.49). In an analysis adjusted for age, body mass index, systolic blood pressure, total cholesterol, high-density lipoprotein cholesterol, smoking status, history of type 2 diabetes, history of coronary heart disease, and alcohol consumption, the HR (95% CI) for AS was 1.39 (1.10–1.76) per 10 nmol/L increase in serum testosterone. When alcohol consumption was replaced with physical activity, the HR (95% CI) was 1.38 (1.09–1.74). Comparing the bottom versus top third of serum testosterone, the corresponding (adjusted) risk estimates were 1.76 (1.11–2.81) and 1.76 (1.10–2.80), respectively. In middle-aged and older Finnish men, elevated levels of serum testosterone were associated with an increased risk of AS. Further research is needed to replicate these findings and assess any potential relevance of serum testosterone in AS prevention.

## 1. Introduction

Aortic stenosis (AS) is well documented to be the most common heart valve disease and it is associated with an increased risk of comorbidities, including infective endocarditis and pulmonary hypertension, as well as mortality [1]. Aortic stenosis, being a vascular disease, shares certain risk factors with atherosclerotic cardiovascular disease (ASCVD), such as male gender, smoking, dyslipidaemia, elevated lipoprotein(a) levels, active inflammation, and diabetes [2,3]. Additionally, the prevalence of AS increases with age, indicating that the growing global aging population will contribute to a rise in the public health burden of AS [4]. Thus, research on underlying risk factors needs to be conducted in large populations. While known risk factors may explain some of the AS risk, their role and contribution in the development of AS remains unclear. Furthermore, a proportion of the risk of AS could be explained by yet-to-be-identified risk factors. Hence, the identification of novel modifiable risk factors that could aid in the development of effective preventive strategies warrants considerable attention.

Testosterone, the principal sex hormone in men, has been investigated as an emerging cardiovascular risk factor for decades, given the increased risk of CVD in men [5]. With increasing age, the levels of testosterone decline in men, but the risk of CVD and mortality increases. Hence, one would expect an inverse relationship between testosterone and CVD risk. However, observational and interventional studies have mostly reported conflicting data on the relationship of endogenous and exogenous testosterone with CVD [5]. Nevertheless, a number of observational studies suggest that low levels of testosterone may be associated with an increased risk of coronary heart disease and CVD [6,7]. Given the overall evidence, we hypothesised that a relationship might exist between testosterone and AS risk. To the best of our knowledge, there has been no previous evaluation of the prospective association between testosterone and the risk of AS. The aim of this study was to assess the nature and magnitude of the association between serum testosterone and the risk of AS using a general-population-based cohort of 2577 middle-aged and older men recruited from eastern Finland.

## 2. Materials and Methods

We employed the Kuopio Ischemic Heart Disease (KIHD) prospective cohort study for this analysis. The KIHD study is a general-population-based study comprising of middle-aged to older men aged 42–61 years who were recruited from Kuopio in eastern Finland. The study design and recruitment methods have been reported previously [8,9]. The baseline cohort comprised 2682 men who had baseline assessments performed between March 1984 and December 1989. In the current analysis, complete information on serum testosterone, relevant covariates, and AS events was available for 2577 men. The research protocol was approved by the institutional review board of the University of Eastern Finland, all study participants provided written informed consent, and study procedures were conducted according to the Declaration of Helsinki.

### 2.1. Assessment of Covariates

The assessment of sociodemographic, lifestyle, and medical characteristics has been described previously [9,10]. Self-administered lifestyle and health questionnaires were used to assess prevalent medical conditions and lifestyle characteristics such as smoking, alcohol consumption, and physical activity. Smoking status was categorised into smokers and nonsmokers. A participant was defined as a smoker if he had ever smoked regularly and had smoked cigarettes, cigars, or a pipe within the past 30 days. Alcohol consumption was assessed using the Nordic Alcohol Consumption Inventory and was reported in g/week. The energy expenditure of total physical activity was assessed using the validated KIHD 12-month leisure-time physical activity questionnaire. The questionnaire captures common leisure-time physical activities in Finland. Based on the energy cost of each physical activity type, a metabolic equivalent (MET) score was assigned to its intensity. One metabolic unit corresponds to an energy expenditure of approximately 1 kcal per kilogram of body weight per hour. Body mass index (BMI) was computed as the ratio of weight in kilograms to the square of height in metres. Resting blood pressure was measured on three occasions between 8:00 and 10:00 am following a supine rest of 5 min using a random-zero sphygmomanometer. The mean of all available measurements was utilised for the analysis. Prevalent T2D was defined as a fasting blood glucose level ≥7.0 mmol/L or a clinical diagnosis of diabetes with dietary, oral, or insulin treatment. A history of coronary heart disease (CHD) was defined as previous myocardial infarction, angina pectoris, the use of nitroglycerin for chest pain ≥ once a week, or chest pain. For the measurements of blood biomarkers, participants fasted overnight and abstained from drinking alcohol for at least 3 days and from smoking for at least 12 h before blood samples were taken between 8:00 am and 10:00 am. Serum testosterone (17B-hydroxy-4-androsten-3-one) was determined with a Spectria Testosterone radioimmunoassay kit (Orion Diagnostic, Espoo, Finland) with an assay sensitivity of 0.10 nmol·L [11]. The inter-assay coefficient of variation for testosterone was 12.2%.

### 2.2. Outcomes

We included all AS events that occurred from the study entry through to the end of follow-up, which were identified through computer linkage to the National Hospital Discharge Registry data and a detailed review of hospital records. No losses to follow-up were recorded. Every resident of Finland has a unique personal identifier that is used in registers. All deaths that occurred between the study entry (March 1984 to December 1989) and 31 December 2018 were included. Outcomes were coded according to the Ninth International Classification of Disease (ICD) codes and the Tenth ICD codes. The diagnoses of AS were validated by two physicians.

### 2.3. Statistical Analyses

Baseline data were summarised using descriptive statistics, presented as means (standard deviation, SD), median (interquartile range, IQR), and (N) percentages. Hazard ratios (HRs) with 95% confidence intervals (Cis) were calculated using Cox proportional hazard regression after confirming no major departure from the assumptions of proportionality of hazards using Schoenfeld residuals [12]. To explore a potential nonlinear dose–response relationship between serum testosterone and AS risk, we constructed a restricted cubic spline with knots at the 5th, 35th, 65th, and 95th percentiles of the distribution of serum testosterone in a multivariable-adjusted model. Serum testosterone was modelled as both continuous [10 nmol/L increase] and categorical (tertile) variables. Hazard ratios were progressively adjusted for in three models: (Model 1) age; (Model 2) Model 1 plus body mass index (BMI), systolic blood pressure (SBP), total cholesterol, high-density lipoprotein cholesterol (HDL-C), smoking status, histories of type 2 diabetes (T2D), and CHD; and (Model 3) Model 2 without alcohol consumption but with physical activity. All statistical analyses were conducted using Stata version MP 16 (Stata Corp, College Station, TX, USA).

## 3. Results

The mean (standard deviation (SD)) age of the 2577 men at baseline was 53 (5) years. The mean (SD) for serum testosterone was 21.0 (7.7) nmol/L, with levels ranging from 1.1 to 56.9 nmol/L (Table 1). Those who developed AS at the end of the follow-up period were older, had higher levels of body mass index (BMI) and total cholesterol, and were more likely to have a history of CHD at the study entry (Table 1). During a median (IQR) follow-up of 27.2 (17.7, 31.0) years, a total of 119 AS cases (annual rate 1.94/1000 person-years at risk; 95% CI: 1.62 to 2.32) occurred.

**Figure 1:**
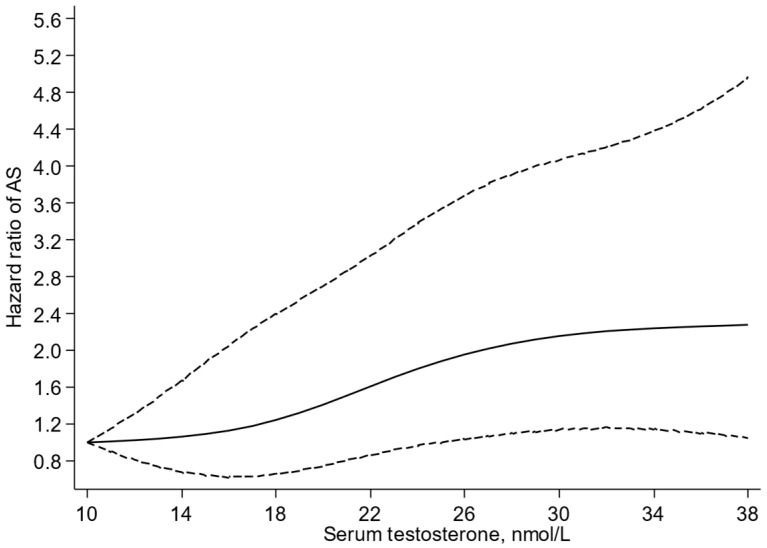
Restricted cubic splines of the hazard ratios of aortic stenosis with serum testosterone. AS, aortic stenosis; reference value for serum testosterone is 10 nmol/L; dashed lines represent the 95% confidence intervals for the spline model (solid line). Models were adjusted for age, body mass index, systolic blood pressure, total cholesterol, high-density lipoprotein cholesterol, smoking status, history of type 2 diabetes, history of coronary heart disease, and alcohol consumption.

A multivariable-restricted cubic spline curve showed that the risk of AS increased in a graded manner with increasing levels of serum testosterone across the range 25–39 nmol/L (*p*-value for nonlinearity = 0.49) (Figure 1). In analysis adjusted for age, BMI, SBP, total cholesterol, HDL-C, smoking status, history of T2D, history of CHD, and alcohol consumption, the HR (95% CI) for AS was 1.39 (1.10–1.76) per 10 nmol/L increase in serum testosterone (Table 2). When alcohol consumption was replaced with physical activity, the HR (95% CI) was 1.38 (1.09–1.74) (Table 2). Comparing the bottom versus top third of serum testosterone, the corresponding HRs (95% CIs) were 1.76 (1.11–2.81) and 1.76 (1.10–2.80), respectively.

## 4. Discussion

Novel findings based on a general-population cohort of middle-aged and older Finnish men indicated that increased levels of serum testosterone were associated with an increased risk of AS, independently of several established cardiovascular risk factors. Furthermore, the risk of AS increased in a graded manner with increasing levels of serum testosterone across the range 25–39 nmol/L, with no evidence of a threshold value.

Given this is the first prospective cohort study to explore the association between serum testosterone and AS risk, the findings cannot be directly compared. However, observational cohort studies have reported on the associations of circulating testosterone with other cardiovascular outcomes. In a prospective population-based study of 2416 Swedish men aged 69 to 81 years, high serum testosterone was reported to be associated with an increased 5-year risk of cardiovascular events [13]. In the population-based Rotterdam study comprising 1032 nonsmoking men and women aged 55 years and over, the authors reported an independent inverse association between levels of testosterone and progression of severe aortic atherosclerosis in men [14]. In a nested case-control study based on the European Prospective Investigation into Cancer in Norfolk (EPIVC-Norfolk), inverse relationships were reported between endogenous testosterone concentrations and CVD and all-cause mortality [15]. Meta-analyses of individual studies have also demonstrated low endogenous testosterone levels to be associated with an increased risk of CVD, CVD mortality, and all-cause mortality [16,17]. Because CVD events tend to occur in older men who also have a higher chronic disease burden, it is not well known if testosterone level has a causal effect on cardiovascular events.

Though the literature is conflicting on the relationship between endogenous testosterone and adverse cardiovascular outcomes, observational evaluations have mostly suggested inverse associations. These associations reflect the beneficial effects of testosterone on the cardiovascular system, which include slowing of atheroma progression, reversal of lipid deposition, lowering of total and low-density lipoprotein levels, improving insulin sensitivity and other measures of glycaemia, lowering of fat mass and adiposity, shortening of QTc intervals, and vasodilation of blood vessels through downregulation of Ca ion channels and upregulation of K ion channels [5]. Considering the evidence, our findings of a graded positive relationship between serum testosterone and AS risk may seem to be at odds with these previous findings. Interestingly, data based on the French Three-City prospective study showed a J-shaped relationship between serum testosterone and risk of ischemic heart disease (IHD) in older men, with individuals in the highest and lowest quintiles having an increased risk of IHD compared with the second quintile [18]. Furthermore, some observational studies and trials of testosterone therapy have reported an increased risk of adverse cardiovascular events associated with testosterone therapy [19]. The mechanistic pathways for the link between elevated serum testosterone and an increased risk of AS are unclear; however, we suggest a number of plausible mechanisms. Testosterone (i) has prothrombotic effects, which increase the risk of stroke and myocardial infarction [5]; (ii) may increase inflammation [20]; and (iii) may increase calcification of arteries. Indeed, it is well documented that calcification in the aortic valve is the final common pathway leading to the development of AS [21]. However, more in vivo translational and mechanistic research is needed to come to a conclusion as to whether testosterone levels are associated with AS.

Though these findings add to the controversy on the role of testosterone in the genesis of adverse cardiovascular outcomes, they do highlight the fact that there might be a possible deleterious role of elevated testosterone in AS development. There is existing research evidence that testosterone should cause significant harm to CVDs. The potential for testosterone therapy to cause CVD events has been a topic of controversy for decades. With several observational studies and trials reporting associations between adverse cardiovascular outcomes and exogenous use of testosterone, the US Food and Drug Administration (FDA) was prompted to issue a safety warning on testosterone therapy for older men in March 2015 [22]. Given the current findings are new and only based on a single observational cohort study, further large-scale studies are warranted in other populations to replicate these results. The Testosterone Replacement Therapy for Assessment of Long-term Vascular Events and Efficacy ResponSE in Hypogonadal Men (TRAVERSE) study [23], a recent clinical trial designed to determine the effect of testosterone therapy on cardiovascular risk, has shed more light on the relationship between testosterone and cardiovascular risk. Despite the enrolment of patients with increased risk for adverse CVD events, the TRAVERSE trial found that testosterone therapy was noninferior to placebo regarding major cardiovascular event risk, although testosterone gel dosing and a mean 22 months of treatment duration were limitations. There was an unexpected higher incidence of pulmonary embolism, atrial fibrillation, and acute kidney injury with testosterone therapy [23]. However, since this trial was not designed to assess AS outcomes, more cohort and intervention studies focussing on AS are also warranted. Testosterone therapy has been shown to improve functional capacity in both men and women with heart failure, reflected by improvements in 6 min walk tests, muscle strength, and cardiorespiratory fitness [24]. Though it is a cause for concern that high levels of serum testosterone may increase the risk of AS, recent studies do suggest that lifestyle factors such as regular physical activity could reduce the risk of AS. We have recently shown that higher cardiorespiratory levels, determined by regular aerobic physical activity [25], are associated with a lower incidence of AS [26].

The strengths of this evaluation include the novelty, the use of a prospective cohort design with long follow-up and zero loss to follow-up, the relatively large sample size comprising a general-population-based sample of middle-aged and older men, and the comprehensive analysis, which included accounting for a comprehensive panel of potential confounders and evaluating the dose–response relationship. We have reliable data on various causes of diseases because disease-specific major outcomes and AS were prospectively ascertained by the Finnish National Discharge Registry. The KIHD study follow-up is 100% due to the fact that, in Finland, CVD events can be detected via the National Hospital Discharge and Death Registry. All of the AS diagnoses, which were combined with baseline data from KIHD, were set in a clinic by experienced doctors. There are limited non-invasive treatment options for AS. While invasive treatment options such as transcatheter aortic valve implantation (TAVI) or open-heart valve surgery are effective, they are expensive and quite demanding. There are several limitations which deserve consideration. First, there were no data on the aetiology and severity of AS as well as the use of common AS interventions such as TAVI or open-heart valve surgery, which precluded a detailed evaluation of the relationship between serum testosterone and risk of AS. Furthermore, AS diagnoses were based on echocardiographic examination. The findings were based on middle-aged and older Finnish men, and hence cannot be generalised to other populations. Participants were middle-aged at the beginning of the study and were followed up over a median duration of 27 years, and, at the end of the follow-up, all participants were over the age of 65 years. This study was initially conducted in a representative sample of middle-aged male population from eastern Finland, an area known for its high prevalence and incidence of atherosclerotic vascular diseases. Therefore, the main interest was to study the risk factors of CVDs in middle-aged men. Unfortunately, we have no available corresponding data among the female population. There are no large prospective studies that would have clearly shown the role of both gender-related hormones in the etiology and development of AS. Though we adjusted for several established and emerging risk factors, the low event rate for AS precluded detailed adjustment. As with observational study designs, reverse causation and residual confounding due to unknown or unmeasured confounding remains a potential alternative explanation for our findings. Given the use of single-baseline values of serum testosterone and the long-term follow-up of the cohort, there was a potential for underestimating the association due to the phenomenon of regression dilution bias.

## 5. Conclusions

In middle-aged and older Finnish men, elevated levels of serum testosterone are associated with an increased risk of AS, consistent with a graded dose–response relationship. Further research is needed to replicate these findings and assess any potential relevance of serum testosterone in AS prevention.

## Figures and Tables

**Table 1:** Baseline participant characteristics overall and according to aortic stenosis development.

	Overall (N = 2577) Mean (SD) or Median (IQR)	No AS (N = 2458) Mean (SD) or Median (IQR)	Developed AS (N = 119) Mean (SD) or Median (IQR)
Testosterone (nmol/L)	21.0 (7.7)	20.9 (7.7)	22.4 (7.8)
*Questionnaire/Prevalent conditions*			
Age at survey (years)	53 (5)	53 (5)	54 (4)
Alcohol consumption (g/week)	31.8 (6.3, 91.5)	32.0 (6.3, 92.3)	30.7 (6.2, 87.7)
History of type 2 diabetes, n (%)	104 (4.0)	99 (4.0)	5 (4.2)
Current smoking, n (%)	814 (31.6)	780 (31.7)	34 (28.6)
History of CHD, n (%)	649 (25.2)	610 (24.8)	39 (32.8)
*Physical measurements*			
BMI (kg/m^2^)	26.9 (3.6)	26.8 (3.4)	27.3 (3.5)
SBP (mmHg)	134 (17)	134 (17)	135 (16)
DBP (mmHg)	89 (11)	89 (11)	88 (10)
Physical activity (KJ/day)	1204 (630, 2000)	1204 (631, 2005)	1202 (605, 1883)
*Blood-based markers*			
Total cholesterol (mmol/L)	5.91 (1.08)	5.90 (1.08)	6.05 (1.11)
HDL-C (mmol/L)	1.29 (0.30)	1.29 (0.30)	1.28 (0.34)

AS, aortic stenosis; BMI, body mass index; CHD, coronary heart disease; CI, confidence interval; DBP, diastolic blood pressure; GFR, glomerular filtration rate; HDL-C, high-density lipoprotein cholesterol; IQR, interquartile range; SD, standard deviation; SBP, systolic blood pressure.

**Table 2:** Association of serum testosterone with aortic stenosis.

Testosterone (nmol/L)	Events/ Total	Model 1		Model 2		Model 3	
		HR (95% CI)	*p*-Value	HR (95% CI)	*p*-Value	HR (95% CI)	*p*-Value
Per 10 nmol/L increase	119/2577	1.26 (1.01–1.57)	0.041	1.39 (1.10–1.76)	0.006	1.38 (1.09–1.74)	0.007
T1 (1.1–17.0)	34/864	ref		ref		ref	
T2 (17.1–23.5)	35/861	1.00 (0.62–1.60)	0.99	1.07 (0.66–1.74)	0.77	1.07 (0.66–1.74)	0.78
T3 (>23.5)	50/852	1.49 (0.97–2.31)	0.072	1.76 (1.11–2.81)	0.017	1.76 (1.10–2.80)	0.018

CI, confidence interval; HR, hazard ratio; ref, reference; T, tertile. Model 1: Adjusted for age. Model 2: Model 1 plus body mass index, systolic blood pressure, total cholesterol, high-density lipoprotein cholesterol, smoking status, history of type 2 diabetes, history of coronary heart disease, and alcohol consumption. Model 3: Model 1 plus body mass index, systolic blood pressure, total cholesterol, high-density lipoprotein cholesterol, smoking status, history of type 2 diabetes, history of coronary heart disease, and physical activity.

## Data Availability

The data that support the findings of this study are not openly available due to reasons of sensitivity and are available from the corresponding author upon reasonable request.
